# Functional Promoter Polymorphisms Govern Differential Expression of HMG-CoA Reductase Gene in Mouse Models of Essential Hypertension

**DOI:** 10.1371/journal.pone.0016661

**Published:** 2011-01-31

**Authors:** Parshuram J. Sonawane, Bhavani S. Sahu, Binu K. Sasi, Parimala Geedi, Govinda Lenka, Nitish R. Mahapatra

**Affiliations:** Cardiovascular Genetics Laboratory, Department of Biotechnology, Indian Institute of Technology Madras, Chennai, India; Brigham and Women's Hospital, United States of America

## Abstract

3-Hydroxy-3-methylglutaryl-coenzyme A [HMG-CoA] reductase gene (*Hmgcr*) is a susceptibility gene for essential hypertension. Sequencing of the *Hmgcr* locus in genetically hypertensive BPH (blood pressure high), genetically hypotensive BPL (blood pressure low) and genetically normotensive BPN (blood pressure normal) mice yielded a number of single nucleotide polymorphisms (SNPs). BPH/BPL/BPN *Hmgcr* promoter-luciferase reporter constructs were generated and transfected into liver HepG2, ovarian CHO, kidney HEK-293 and neuronal N2A cells for functional characterization of the promoter SNPs. The BPH-*Hmgcr* promoter showed significantly less activity than the BPL-*Hmgcr* promoter under basal as well as nicotine/cholesterol-treated conditions. This finding was consistent with lower endogenous *Hmgcr* expression in liver and lower plasma cholesterol in BPH mice. Transfection experiments using 5′-promoter deletion constructs (strategically made to assess the functional significance of each promoter SNP) and computational analysis predicted lower binding affinities of transcription factors c-Fos, n-Myc and Max with the BPH-promoter as compared to the BPL-promoter. Corroboratively, the BPH promoter-luciferase reporter construct co-transfected with expression plasmids of these transcription factors displayed less pronounced augmentation of luciferase activity than the BPL construct, particularly at lower amounts of transcription factor plasmids. Electrophoretic mobility shift assays also showed diminished interactions of the BPH promoter with HepG2 nuclear proteins. Taken together, this study provides mechanistic basis for the differential *Hmgcr* expression in these mouse models of human essential hypertension and have implications for better understanding the role of this gene in regulation of blood pressure.

## Introduction

Essential hypertension, the chief risk factor for cardiovascular and renal diseases, is often associated with and complicated by dyslipidemia [1], [2]. 3-Hydroxy-3-methylglutaryl-coenzyme A [HMG-CoA] reductase gene (*Hmgcr/HMGCR*) is a candidate gene for hypertension; it translates to the rate-limiting enzyme in the cholesterol biosynthesis pathway and cholesterol is the precursor of glucocorticoid steroid hormones that play a profound role in blood pressure homeostasis and hypertension [3]–[7]. Consistently, the G allele of *HMGCR* rs17238540 (G/T) single nucleotide polymorphism [SNP] was associated with higher blood pressure [BP] and higher stroke risk in an European population of ∼23,000 participants [8]. Moreover, this SNP was associated with the BP response to urinary sodium: potassium ratio [9] and response to statin (inhibitor of HMGCR enzyme) therapy in terms of total cholesterol and triglyceride lowering [10]. Two common and tightly linked *HMGCR* SNPs were also significantly associated with reduced efficacy of pravastatin therapy [11]. Additionally, investigations on gene expression pattern in adrenal glands of two independent, inbred, homozygous rodent models of human essential hypertension (viz. spontaneously hypertensive rat and blood pressure high [BPH] mice) revealed ∼2- to 3-fold over-expression of *Hmgcr* in these strains as compared to their corresponding controls (viz. Wistar/Kyoto rat and blood pressure low [BPL] mice) [12], [13]. These findings suggested the possibility that an altered *Hmgcr* expression might be a systematic facet of hereditary hypertension in mammals, perhaps even contributing to diverse metabolic abnormalities associated with this common disorder. However, molecular basis of the differential *Hmgcr* expression in these animal models has not been studied. *Hmgcr* levels in other tissues (e.g., liver) of BPL and BPH mice also remain unknown.

The hypertensive mouse strain BPH was developed in a breeding program based solely on selection by elevated BP and it parallels human hypertension [14]. The BPH strain exhibits many of the co-morbidities observed in human hypertension, such as higher heart rate, larger hearts and kidneys, higher left ventricular weight and early mortality than the hypotensive BPL strain [14]. During generation of the BPH and BPL strains, the normotensive inbred strain BPN (blood pressure normal) was derived from the unselected control population and this strain serves as a control for hypertensive BPH and hypotensive BPL mice [14].

In the present study, we sequenced the mouse *Hmgcr* locus (proximal promoter, 20 exons and flanking intronic regions) in the BPH, BPL and BPN strains and discovered several SNPs in promoter and coding exonic regions. Next, we assessed the quantitative impact of the promoter SNPs on *Hmgcr* gene expression by computational as well as experimental analyses. The results revealed that two promoter SNPs (C-874T and C-740T) altered binding affinities of several transcription factors (n-Myc, Max and c-Fos) and modulated *Hmgcr* expression in these mouse models of human essential hypertension.

## Materials and Methods

### Ethics Statement/study approval

The present study was approved by the Institutional Biosafety Committee at Indian Institute of Technology Madras (IIT Madras), Chennai in June 2008.

### Mouse strains and tissue samples

Liver tissue samples from 5–7 weeks old male BPH (strain BPH/2J, at inbred generation F66) and BPL (strain BPL/1J, at inbred generation F65) mice were collected in RNAlater® solution (Ambion, USA) at the Jackson Laboratory (Bar Harbor, USA; www.jax.org) and shipped to our laboratory following institutional norms. BPH males display ∼120 mm systolic BP (SBP) while BPL males display ∼70 mm SBP at 4–15 weeks of age [14]. At 21 weeks of age, the SBP of BPH mice increases further to ∼130 mm while that of BPL remains almost unchanged [14]. We chose 5 to 7 weeks old mice for measurement of *Hmgcr* gene expression levels because at that early age, BPH mice did not attain the maximal elevation of BP. Therefore, studying these mice might allow us to minimize the effects of aging-related confounding factors on *Hmgcr* gene expression and increase the chance of detecting pathogenic role for *Hmgcr* in hypertension.

### Extraction of RNA and real-time PCR

Total RNA samples from liver tissues of four BPH and four BPL mice were isolated using the TRIZOL reagent (Invitrogen, USA). RNA concentrations were estimated by UV-spectrophotometry (Eppendorf Biophotometer, Germany) and the integrity of RNA molecules was assessed from the appearance of 28S and 18S bands on agarose gels.

RNA samples were subjected to total cDNA synthesis by using the ProtoScript Moloney Murine Leukemia Virus [M-MuLV] Taq RT-PCR kit (New England Biolabs, USA) and the absence of genomic DNA contamination was ascertained. See Text S1 for details.

Real-time PCR was carried out using the DyNAmo™ HS SYBR® Green qPCR Kit (Finnzymes, USA) and following *Hmgcr* gene specific primers: forward, [+11763 bp] 5′-CCCTGAGTTTAGCCTTCCTTTTG-3′ [+11786 bp] and reverse, [+11880 bp] 5′-GCTTTCTTTGAGGTCACGACGG-3′ [+11858 bp]. For normalization of *Hmgcr* expression, GAPDH and 18S rRNA abundances were measured using the following primer pairs: GAPDH forward, 5′-CCTCGTCCCGTAGACAAAATG-3′ and GAPDH reverse, 5′-TGAAGGGGTCGTTGATGGC-3′ [15]; 18S forward, 5′-GTAACCCGTTGAACCCCATT -3′ and 18S reverse, 5′-CCATCCAATCGGTAGTAGCG-3′ [16]. See Text S1 for details. The relative gene expression levels were determined by calculating the 2^(−ΔΔCt)^ values [17].

### Sequencing of mouse *Hmgcr* gene for polymorphism discovery

**Figure 1:**
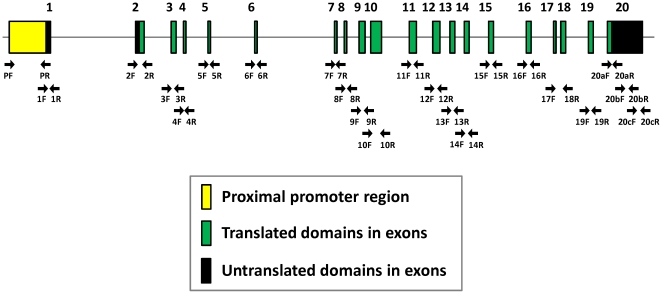
Schematic structure of the mouse *Hmgcr* gene. Exon/intron structure of the mouse *Hmgcr* gene (RefSeq NM_008255, from the UCSC genome browser). The upstream ∼1 kb promoter region and 20 exons spanning ∼21.5 kb region of mouse chromosome 13 are shown. The exon/intron lengths are not drawn to scale. Locations of the primers used for PCR-amplification and sequencing of *Hmgcr* genomic regions (upstream promoter, exons, UTRs and exon-intron boundaries) of BPH, BPL and BPN mice are indicated by arrows. Sequences of the primers are given in Table 1.

Genomic DNA samples of BPL/1J, BPH/2J and BPN/3J mice were obtained from the Jackson laboratory (Bar Harbor). Primers were designed using the mouse *Hmgcr* reference sequence NM_008255.2 (from the UCSC Genome Browser) to amplify ∼1 kb promoter region, each of the 20 exons as well as 50–100 bp of flanking intronic regions (Table 1 and Fig. 1). PCR was carried out using Phusion™ High-Fidelity DNA polymerase and dNTPs from New England Biolabs, USA. Agarose-gel purified PCR products served as templates for sequencing, with the exception that purified PCR products inserted into the promoterless pGL3-Basic vector (Promega, USA) were used for sequencing the promoter region. DNA samples were sequenced using ABI 3130 Genetic Analyser and BigDye Terminator Cycle Sequencing Ready Reaction Kit (Applied Biosystems, USA).

**Table 1:** List of primers used for PCR amplification and sequencing of the *Hmgcr* gene in BPL, BPH and BPN mouse strains.

Target	Forward Primer (nucleotide positions*)	Reverse Primer (nucleotide positions*)	Product size (bp)
Promoter	PF (−961/−940): 5′-CGGGGTACCTAAAGTGGGTAGGTATATCCGG-3′	PR (24/43): 5′-CCGCTCGAGCTCACCTCCGGATCTCAATG-3′	1004
Exon 1	1F (−90/-70): 5′-CGGACGATCCTTCCTTATTGG-3′	1R (361/383): 5′-TTTGCAGCCTACATCTCCATCAC-3′	473
Exon 2	2F (3989/4008): 5′-AAGAAGTGGCAAGCACCGTG-3′	2R (4615/4593): 5′-GAGAAAGCGTTCAAACAAGGACC-3′	627
Exon 3	3F (4215/4238): 5′-TGGGAAGTTATTGTGGGAACAGTG-3′	3R (4783/4762): 5′-CTGAAATCCAAAGTCTGCCAGC -3′	569
Exon 4	4F (4798/4822): 5′-AGTGTTGGGTTCATTCAGCAGTTAG-3′	4R (5266/5245): 5′-GGCAAAAAAGACTTGGCACAGC-3′	469
Exon 5	5F (5885/5906): 5′-AGCAGGAAAGTGGTCATGCCAC-3′	5R (6221/6200): 5′-GGGAAATGGGGAAGTGAGACAC-3′	337
Exon 6	6F (7630/7653): 5′-TCATGTAGGACCCAGGATGCTCTC-3′	6R (7947/7923): 5′-CCACACACTTACAATATCCCCGTTC-3′	318
Exon 7	7F (10426/10446): 5′-TTGTGCTGATGCTTGGGTCTG-3′	7R (10886/10867): 5′-ATGGCTGAGCTGCCAAATTG-3′	461
Exon 8	8F (10655/10674): 5′-TCGGCTGCATGTCAGTGTTG-3′	8R (11030/11010): 5′-CAAGCAAAAGCCCCCAAATAC-3′	376
Exon 9	9F (11315/11338): 5′-GGATGTTTGAACCCTTACAGCACC-3′	9R(11924/11903): 5′-TCCTTCTCACAAGCAGAGGCTC-3′	610
Exon 10	10F (11481/11503): 5′-TTGGTCCTTGTTCACGCTCATAG-3′	10R (12138/12114): 5′-CTCTGCTTGTAGTCTCTGCTTCCAC-3′	658
Exon 11	11F (11982/12004): 5′-CCGTGCTGTGTTCTTCATCTCAG-3′	11R(12345/12322): 5′-TCCTCTGTATTCTCCCCCAGTGTG-3′	364
Exon 12	12F (13928/13949): 5′-TGGGGCATAGTCTGGCTAAGTG-3′	12R (14412/14389): 5′-GAGCAAGCAAACAAACTGTTGGAC-3′	485
Exon 13	13F (14663/14684): 5′-CACTGATGAAGCCCTTGGTTTG-3′	13R (15088/15067): 5′-CTTGTGTCTGTCCCCGAATCTG-3′	426
Exon 14	14F (14982/15003) FP: 5′-GCAGAGCCATAAGCGTGAGTTG-3′	14R (15546/15523): 5′-CTTTTACCTGCTGAGCCACCTTAC-3′	565
Exon 15	15F (15812/15832): 5′-TCCATGCCTGAGAATGCCTTC-3′	15R(16154/16131): 5′-GGGTACGGTAGCACAGTTATGGTC-3′	343
Exon 16	16F (17947/17969): 5′-ACGGCTGCGTTACAACTGTTAAG-3′	16R (18336/18313): 5′-ATACCCACCCTGGCTTCAGACTTC-3′	390
Exon 17-18	17F(18535/18559): 5′-AGAGACAGAGCGGTAGATGTGAGTG-3′	18R (19139/19116): 5′-AGTCCCTGCCTTAACTTCCCTTAG-3′	605
Exon 19	19F (19609/19629): 5′-TTGCCACCAGCGTTTCTAATG-3′	19R(19942/19921): 5′-TCCAGGGCTTGGACTGAAGTTG-3′	334
	20aF (20136/20156): 5′-CACATTCACATGCACCCCAAC-3′	20aR(20949/20972): 5′-GAGAGGTCCGACTTGCTTGTACTC-3′	837
Exon 20	20bF (20726/20746): 5′-TGCTGGTCTATTGATTGGGGG-3′	20bR (21214/21235): 5′-AACGAGCACTGTCTTCTCTGGC-3′	510
	20cF (20895/20918): 5′-GCTGTGCCACACTCTGCACTAAAG-3′	20cR (21603/21626): 5′-TGACACAATCACTAAGAGGCTCCC-3′	696

*The nucleotides were numbered upstream (−) or downstream (+) of the cap site.

### Construction of *Hmgcr* promoter-reporter plasmids

Approximately 1 kb *Hmgcr* promoter region was PCR-amplified from BPH/BPL/BPN genomic DNA sample using the following primers: forward, 5′-CGG**GGTACC**TAAAGTGGGTAGGTATATCCG-3′ and reverse, 5′-CCG**CTCGAG**CTCACCTCCGGATCTCAATGG-3′ (with added *Kpn*I and *Xho*I sites at 5′ ends in forward and reverse primers respectively, shown in bold). The amplified promoter fragments were inserted between *Kpn*I and *Xho*I sites in the firefly luciferase reporter vector pGL3-Basic (Promega). Resulting plasmids were named as BPH-961, BPL-961 and BPN-961, which contained −961 bp to +43 bp region of BPH, BPL and BPN *Hmgcr* (numberings are with respect to the 1^st^ nucleotide of Exon 1 as +1). Similarly, the promoter-reporter plasmids BPH-769, BPL-769 and BPN-769 (harbouring −769 bp to +43 bp region) were generated by insertion of PCR-amplified products in the pGL3-basic vector using the following primers: forward, 5′-CGG**GGTACC**AAACGCCAGAAGCAGAAGGTG-3′ and reverse, 5′-CCG**CTCGAG**CTCACCTCCGGATCTCAATGG-3’ (with added *Kpn*I and *Xho*I sites in forward and reverse primers respectively, shown in bold). We also constructed the promoter reporter plasmids BPH-651, BPL-651 and BPN-651 (containing −651 bp to +43 bp region) by digestion of the BPH-961, BPL-961 and BPN-961 constructs with *Kpn*I and *Eco*RI (the *Eco*RI site is located at −652/−647 bp position in the *Hmgcr* promoter), excision of ∼5.4 kb fragment from gel, treatment with Mung Bean nuclease (New England Biolabs) to remove overhangs and re-circularization with T4 DNA ligase (New England Biolabs). The correct insertion/orientation and existence of SNPs in cloned DNA fragments were confirmed by sequencing of the entire inserts in several clones. The plasmids were purified on columns using an endotoxin-free plasmid DNA purification kit (Hi-Media, India) for transfection experiments.

### Cell culture, transfection and reporter assay

Human hepatic cell line HepG2, Chinese hamster ovarian cell line CHO, human embryonic kidney cell line HEK-293 and mouse neuroblastoma cell line N2A were obtained from the National Center for Cell Sciences, Pune, India. Cells were cultured in Dulbecco's Modified Eagle's Medium (DMEM) with high glucose and GlutaMAX™-I (Invitrogen), supplemented with 10% fetal bovine serum (Invitrogen), penicillin G (100 U/ml) and streptomycin sulfate (100 µg/ml) (Invitrogen) at 37°C with 6% CO_2_. HepG2 and N2A cells (grown at 50–60% confluence in 12-well plates) were transfected with 1 µg/well of promoter-reporter plasmids using Lipofectamine-2000 (Invitrogen). Similarly grown CHO and HEK-293 cells were transfected with 2 µg/well of plasmids by calcium phosphate method [18]. As an internal control for transfection efficiency, cells were co-transfected with 0.5 µg/well of a β-galactosidase expression plasmid. Cells were lysed 24–30 hours after transfection for reporter assays. The luciferase assay was carried out with some modifications of a previously described method [19], [20]. The beta-gal assay was carried out using ortho-nitrophenyl β-D-galactopyranoside as substrate. See Text S1 for details.The results were expressed as firefly luciferase/β-galactosidase activity.

To test the effect of nicotine on *Hmgcr* promoter activity, HepG2 and CHO cells transfected with promoter-reporter plasmids were treated with various doses (100 µM to 1 mM) of nicotine bitartarate (Sigma-Aldrich, USA) five hours after transfection and incubated for 16–18 hrs. Likewise, to test the effect of cholesterol, cells were grown in lipid-free DMEM medium (Hyclone-Thermo, USA) and transfected cells were treated with 0.5 µg/ml of 25-hydroxycholesterol and 12 µg/ml cholesterol (Sigma-Aldrich) for 24–30 hrs. Cells were treated with this mixture of sterols because although 25-hydroxycholesterol was more potent than cholesterol in suppressing reductase activity, but it could not replace cholesterol in maintaining the cell growth [21], [22]. Cells were lysed and assayed for luciferase activity as described above.

In some experiments, promoter-reporter constructs were co-transfected with various transcription factor plasmids into CHO cells: pmiw-nMyc expressing mouse n-Myc cDNA [23], pmiw-Max expressing human Max cDNA [24] and pc-Fos expressing mouse c-Fos cDNA [25]. In these co-transfection experiments, the insert-free vectors pmiwSV (in case of n-Myc/Max) and pSGI (in case of c-Fos) were used as balancing plasmids in different transfection mixtures. As a control for varying cell number within individual wells, total protein contents were measured in cell lysates using Bradford's assay reagent (Sigma-Aldrich). Luciferase activities in cell lysates were expressed as relative light units [RLU]/µg protein.

### Electrophoretic mobility shift assay (EMSA)

Nuclear protein extracts from HepG2 cells were prepared using the ProteoJET cytoplasmic and nuclear protein extraction kit (Fermentas Life Sciences, USA) and stored in aliquots at −80°C until use. See Text S1 for details.

The following oligos and their complementary strands were obtained from Ocimum Biosolutions, India: BPH-nMyc/Max, 5′-GTGTAAGCACC**C**GAGAGTGGGA-3 (harboring C alelle at the −740 bp position, shown in bold); BPL-nMyc/Max, 5′- GTGTAAGCACC**T**GAGAGTGGGA -3 (harboring T allele at the −740 bp position, shown in bold); BPH-c-Fos, 5′-GAAGGGTAAGTTACTC**C**AGGCTAACA-3′ (harboring C allele at the -874 bp position, shown in bold); BPL-c-Fos, 5′- GAAGGGTAAGTTACTC**T**AGGCTAACA-3′ (harboring T allele at the −874 bp position, shown in bold) and the control primers nMyc/Max-consensus, 5′- GTGTAAG**CACGTG**AGAGTGGGA -3′ (consensus n-Myc motif in bold) and c-Fos-consensus, 5′-GAAGGGTAA**GTGAGTCAA**GGCTAACA-3′ (consensus c-Fos motif in bold). These single stranded oligomers were biotinylated using the Biotin 3′ End Labeling kit (Pierce, USA) and annealed. See Text S1 for details.

For EMSA, 10 µg of nuclear protein extract was incubated with binding buffer [10 mM Tris, 50 mM KCl and 1 mM dithiothreitol at pH 7.5], 50 ng/µl poly-dI-dC and 20 fmol of biotinylated oligo for 20 min at room temperature. The reaction mixtures were resolved on 1.5 mm thick 5% non-denaturing polyacrylamide gels and transferred to nylon-66 membranes (Fluka, USA). The DNA oligomers were UV cross-linked to membrane at 312 nm for 10 min. The biotinylated probes were detected by chemiluminescence using LightShift Chemiluminescent EMSA kit (Pierce).

### Data presentation and statistics

Promoter/reporter transient transfections were carried out at least three times and results were expressed as mean ± S.E. Statistical significance was calculated by student's t-test and one-way analysis of variance (ANOVA) with Tukey-Kramer multiple comparisons post-test, as appropriate in different experiments using the InStat 3 program (GraphPad software, USA).

## Results

### Discovery of polymorphisms in the mouse *Hmgcr* gene

**Figure 2:**
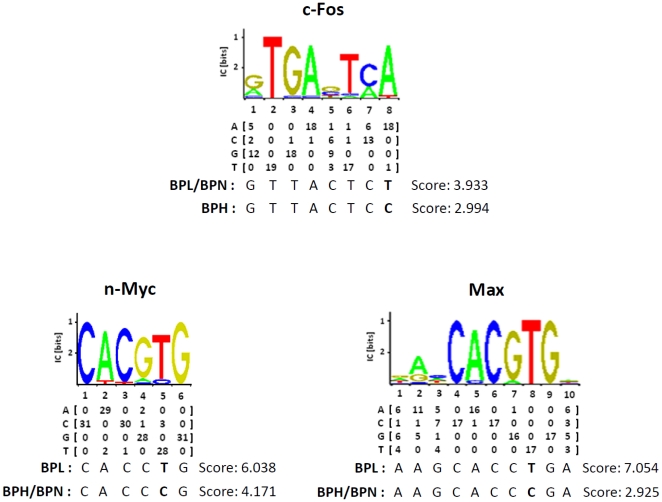
*Hmgcr* promoter-SNPs alter potential binding affinities of promoter motifs with putative transcription factors. Pictorial presentations as well as numerical nucleotide matrixes for c-Fos, n-Myc and Max binding motifs according to ConSite (http://asp.ii.uib.no:8090/cgi-bin/CONSITE/consite) are shown. The −881 to −874 bp region of the mouse *Hmgcr* promoter contains a putative binding site for c-Fos while the −744 to −739 bp and −747 to −738 bp regions contain putative binding sites for n-Myc and Max respectively. The T→C single nucleotide polymorphism (SNP) at −874 bp alters the potential binding affinity of c-Fos to the promoter motif in BPL/BPN versus BPH, the ConSite scores being 3.933 *vs.* 2.994 (top panel). The T→C SNP at −740 bp alters the potential binding affinity of n-Myc to the promoter motif in BPL versus BPN/BPH, the ConSite scores being 6.038 *vs.* 4.171 (bottom panel, left). The T→C SNP at −740 bp also alters the potential binding affinity of Max to the promoter motif in BPL versus BPN/BPH, the ConSite scores being 7.054 *vs.* 2.925 (bottom panel, right).

Sequencing of the *Hmgcr* locus in BPH, BPL and BPN mice yielded several SNPs (Table 2). In the promoter region, 3 SNPs were detected: at −874 bp (C/T), −740 bp (C/T) and −486 bp (T/ΔT). Analysis of the sequences immediately surrounding these promoter SNPs by ConSite (http://asp.ii.uib.no:8090/cgi-bin/CONSITE/consite; [26]) for identification of cis-regulatory elements revealed the presence of putative binding sites for the transcription factors c-Fos (at -881 to −874 bp), n-Myc (at −744 to −739 bp) and Max ( = Myc-associated factor X; at −747 to −738 bp). The −874T variant in BPL/BPN contributed to a better binding site than the −874C variant in BPH for c-Fos (Fig. 2). Likewise, the −740T allele in BPL contributed to better binding sites for n-Myc and Max than the −740C allele in BPN/BPH (Fig. 2). The deletion of T at −486 bp in BPH did not alter any transcription factor binding site.

**Table 2:** Polymorphisms in the *hmgcr* gene in mouse models of essential hypertension.

Location of the SNP(nucleotide position, region)*	Mouse strains			Functional implication (alteration of transcription factor binding affinity#/amino acid residue)
	BPN	BPH	BPL	
−874 bp, promoter	T	C	T	Binding affinity of c-Fos is more for “T” as compared to “C”
−740 bp, promoter	C	C	T	Binding affinity of n-Myc and Max is more for “T” than “C”
−486 bp, promoter	T	ΔT	T	Deletion of one T from a 13 nucleotide polyT region
+4643 bp, exon 3	T	A	A	Alteration of the amino acid Phenyl alanine (BPN) to Isoleucine (BPH and BPL) in the trans-membrane region of the protein (at 62 residue position)
+4676 bp, exon 3	T	A	A	Alteration of the amino acid Phenyl alanine (BPN) to Isoleucine (BPH and BPL), at the 73 residue position, in the trans-membrane region of the protein
+11531 bp, exon 9	A	G	G	No change in the amino acid residue
+11577 bp, exon 9	A	A	G	Alteration of the amino acid Asparagine (BPN and BPH) to Aspartate (BPL) at the 296 residue position, in the trans- membrane region of the protein
+12264 bp, exon 11	G	A	G	Alteration of the amino acid Glutamic acid (BPN and BPL) to Lysine (BPH) at the 455 residue position, in the catalytic region of the protein
+15984 bp, exon 15	T	G	G	Alteration of the amino acid Leucine (BPN) to Arginine (BPH and BPL) at the 645 residue position, in the catalytic region of the protein
+18745 bp, exon 17	G	T	T	Alteration of the amino acid Glycine (BPN) to Cysteine (BPH and BPL) at the 763 residue position, in the catalytic region of the protein
+18849 bp, exon 18	G	T	T	Alteration of the amino acid Lysine (BPN) to Asparagine (BPH and BPL) at the 770 residue position, in the catalytic region of the protein
+21205 bp, exon 20 (3′-UTR)	T	C	C	-
+21410 bp, exon 20 (3′-UTR)	T	C	C	-
+21446 bp, exon 20 (3′-UTR)	T	C	C	-

*The numbering of the polymorphisms was done considering the first nucleotide of the Exon 1 as +1.

# The putative transcription factor binding sites and the binding affinity towards the promoter sequences harboring the polymorphisms were predicted using the CONSITE web tool (http://asp.ii.uib.no:8090/cgi-bin/CONSITE/consite/).

In addition to the above-mentioned promoter variations, eight SNPs in coding exons and three SNPs in the 3′-UTR were detected (Table 2). Seven of the exonic SNPs altered amino acid residues and four of those belonged to the catalytic domain of the Hmgcr enzyme. Of note, no SNP was detected in the 5′-UTR region and intronic regions flanking the exons.

### Basal expression of the *Hmgcr* promoters in cultured cells

**Figure 3:**
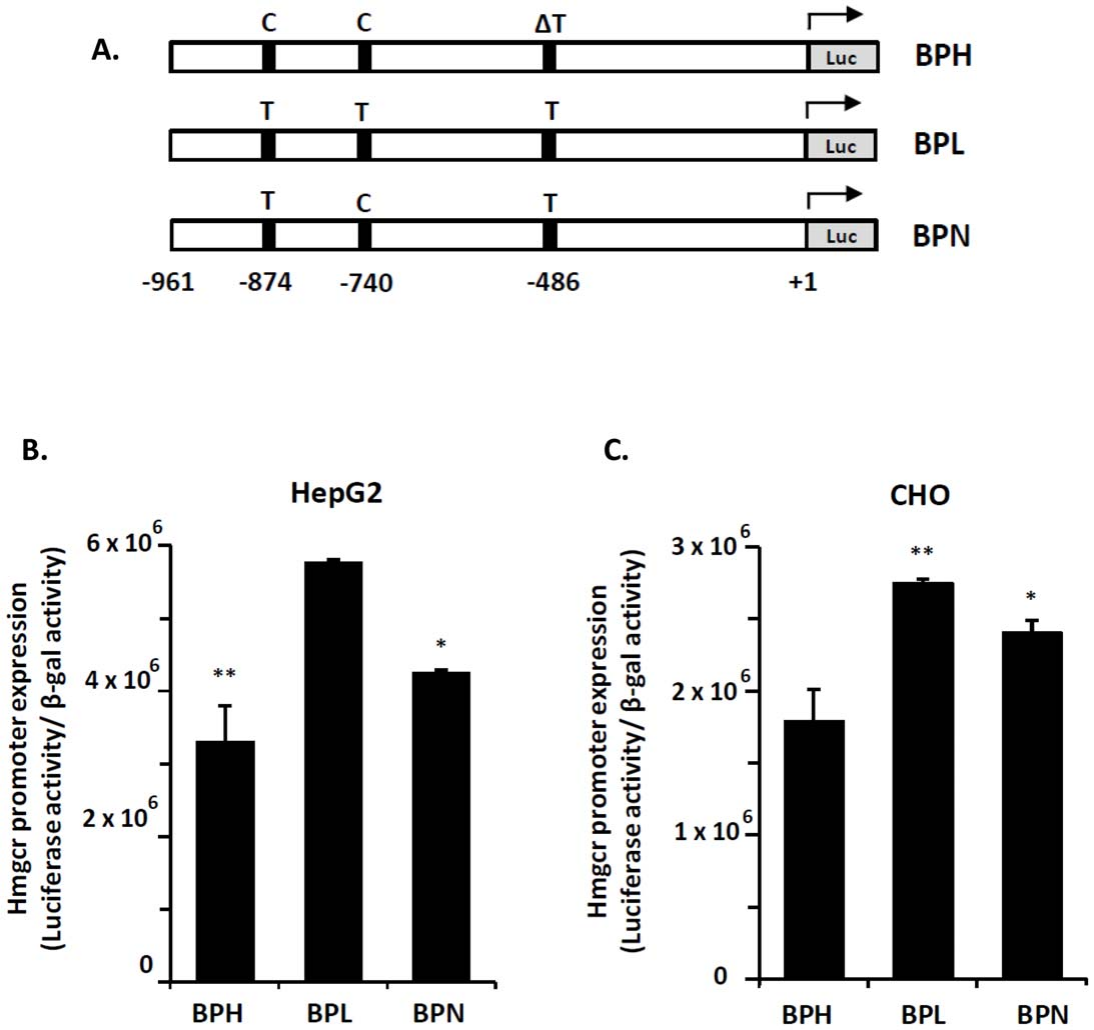
Expression of *Hmgcr* promoter- luciferase reporter plasmids in cultured cells. ***Panel A:*** Schematic presentation of *Hmgcr* promoter-luciferase reporter constructs. The locations of promoter SNPs (at −874, −740 and −486 bp) in BPH, BPL and BPN strains are indicated. ΔT shows the deletion of T and −961 indicates the length of promoter used in this study. ***Panels B and C:*** Comparison of *Hmgcr* promoter strengths among BPH, BPL and BPN mice. Promoter reporter constructs harboring −961 to +43 bp region of BPH/BPL/BPN *Hmgcr* gene were transfected to HepG2 and CHO cells, along with the co-transfected control plasmid pCMV-βGal (β-galactosidase driven by CMV promoter). The cells were assayed for luciferase and β-galactosidase activities 24–30 hrs after transfection. Values shown in the bar graph are the Means ± S.E. of normalized (ratioed) luciferase activity to the β-galactosidase activity from at least three independent experiments. The three strains' transfected promoters displayed significantly different activities in both HepG2 (ANOVA F = 19.955, p<0.01) and CHO (ANOVA F = 12.496, p<0.01) cells as determined by one-way ANOVA with Tukey-Kramer multiple comparisons post-test. In general, the transfected BPL promoter was more active than the BPH promoter while the BPN promoter displayed intermediate expression. (**) and (*) indicate p<0.01 and p<0.05 respectively, compared with BPL in the case of HepG2 cells and compared with BPH in the case of CHO cells.

To test functional implication of the *Hmgcr* promoter SNPs, BPH/BPL/BPN promoter/luciferase reporter constructs (Fig. 3A) were transfected into HepG2, CHO, HEK-293 and N2A cells. In HepG2 cells, the BPL promoter activity was ∼1.8-fold higher (p<0.01) than the BPH promoter; the BPN promoter activity was ∼1.4-fold less (p<0.05) than the BPL promoter (Fig. 3B). Similarly, CHO cells showed ∼1.5-fold higher (p<0.01) promoter activity in the case of BPL than BPH; the BPN promoter was ∼1.3 –fold more active (p<0.05) than the BPH promoter (Fig. 3C). In HEK-293 cells, BPL and BPN promoter activities were ∼1.4-fold (p<0.01) and ∼2.2-fold (p<0.01) respectively higher than the BPH promoter activity (Table S1). In N2A cells, activities of the BPL and BPN promoters were ∼1.9-fold (p<0.01) and ∼1.4-fold (p<0.05) respectively higher than the BPH promoter (Table S1). Thus, across these cell lines, in general, the BPL promoter was more active than the BPH promoter while the BPN promoter expression was intermediate.

### Endogenous *Hmgcr* expression in BPH and BPL mice

**Figure 4:**
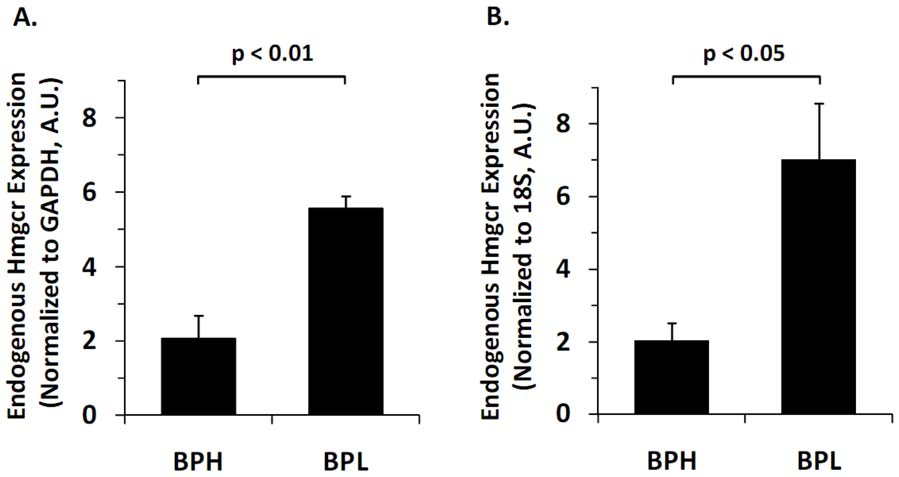
Endogenous *Hmgcr* expression in BPH and BPL liver tissues. Total RNA was extracted from liver tissues of BPH (n = 4) and BPL (n = 4) mice and total cDNA was synthesized. Real-time PCR using the cDNA preparations were carried out with mouse *Hmgcr* specific primers as described in the Materials and Methods. The *Hmgcr* mRNA levels among the samples were normalized by GAPDH (panel A) and 18S rRNA (panel B). The *Hmgcr* mRNA abundance in BPL was significantly higher than BPH.

To study whether endogenous *Hmgcr* expressions differ between BPH and BPL mice in parallel to transfected promoter activities, we measured *Hmgcr* mRNA levels in liver tissues by real-time PCR. We chose liver tissues for this experiment because the liver is the primary site of *de novo* cholesterol biosynthesis and an important regulator of whole-body intermediary metabolism [27], [28]. The BPL liver samples showed significantly higher (∼2.6-fold when normalized to GAPDH, p = 0.002; ∼3.4-fold when normalized to 18S rRNA, p = 0.022) level of *Hmgcr* mRNA than the BPH liver samples (Fig. 4).

### Augmentation of *Hmgcr* promoter activities by nicotinic stumulation

**Figure 5:**
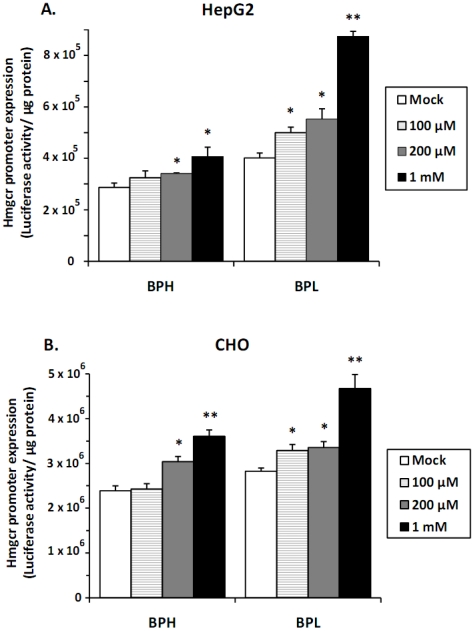
Effect of nicotine on *Hmgcr* promoter expression. HepG2 and CHO Cells were transfected with BPL/BPH *Hmgcr* promoter (−961 to +43 bp)/firefly luciferase construct. Transfected cells were treated with various doses of nicotine (100 µM, 200 µM and 1 mM) and incubated for 16–18 hrs. Cells were lysed and assayed for luciferase activity and protein concentration. Results were expressed as Mean ± S.E. of the ratio of firefly luciferase activity/µg protein. Each experiment was performed in triplicate and repeated at least three times. Nicotine significantly induced the expression of *Hmgcr* promoters in both HepG2 and CHO cells, to a greater extent in the case of BPL than BPH. (**) and (*) indicate p<0.01 and p<0.05 respectively, with respect to the mock (without stimulation).

Since nicotine administration augments cholesterol biosynthesis [29]–[31], we tested the effect of nicotine (100 µM–1 mM) on BPH- and BPL- *Hmgcr* promoters in cultured cells. Acute nicotine treatment caused significantly dose-dependent induction of promoter activities in both HepG2 (up to ∼2.2-fold; Fig. 5A) and CHO cells (up to ∼1.7-fold; Fig. 5B). Similar to basal expressions, BPL-promoter activities after nicotine were greater than BPH at all nicotine doses. At the highest dose (1 mM) the BPL vs. BPH promoter activities were: ∼8.7×10^5^ RLU/µg protein *vs*. ∼4.1×10^5^ RLU/µg protein in HepG2 cells, p<0.01; ∼4.7×10^6^ RLU/µg protein *vs*. ∼3.6×10^6^ RLU/µg protein in CHO cells, p<0.05; (Fig. 5).

### Differential responses of *Hmgcr* promoters to cholesterols

**Figure 6:**
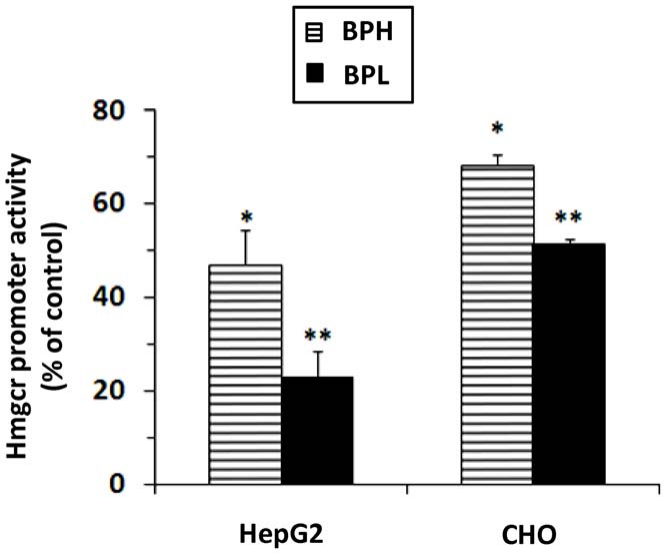
Effect of sterols on *Hmgcr* promoter expression. HepG2 and CHO Cells cultured in lipoprotein-deficient medium were transfected with BPL/BPH *Hmgcr* promoter (−961 to +43 bp)/firefly luciferase construct. Transfected cells were treated with of 25-Hydroxycholeserol (0.5 µg/ml) and of cholesterol (12 µg/ml) and incubated for 24–30 hrs. Cells were lysed and assayed for luciferase activity as well as protein concentration (for normalization). The normalized *Hmgcr* promoter activity in response to sterols (as percentage of control/basal) were expressed as Mean ± S.E. Each experiment was performed in triplicate and repeated at least three times. Significant reduction in promoter activity was observed in both HepG2 and CHO cells, to a greater extent in the case of BPL than BPH. (**) and (*) indicate p<0.01 and p<0.05 respectively, with respect to the mock (without stimulation).

Sterols are known to negatively regulate *Hmgcr* expression as well as enzyme activity [22], [32], [33]. Therefore, we tested the effect of cholesterols on BPH- and BPL- *Hmgcr* promoter activities. Both promoters displayed significant reductions in luciferase activities with respect to corresponding basal values; the extent of down-regulation was more pronounced in the case of BPL than BPH (∼77%, p<0.01 *vs.* ∼53%, p<0.05 in HepG2 cells and ∼49%, p<0.01 *vs.* ∼32%, p<0.05 in CHO cells; Fig. 6).

### Functional characterization of the *Hmgcr* promoter SNPs

**Figure 7:**
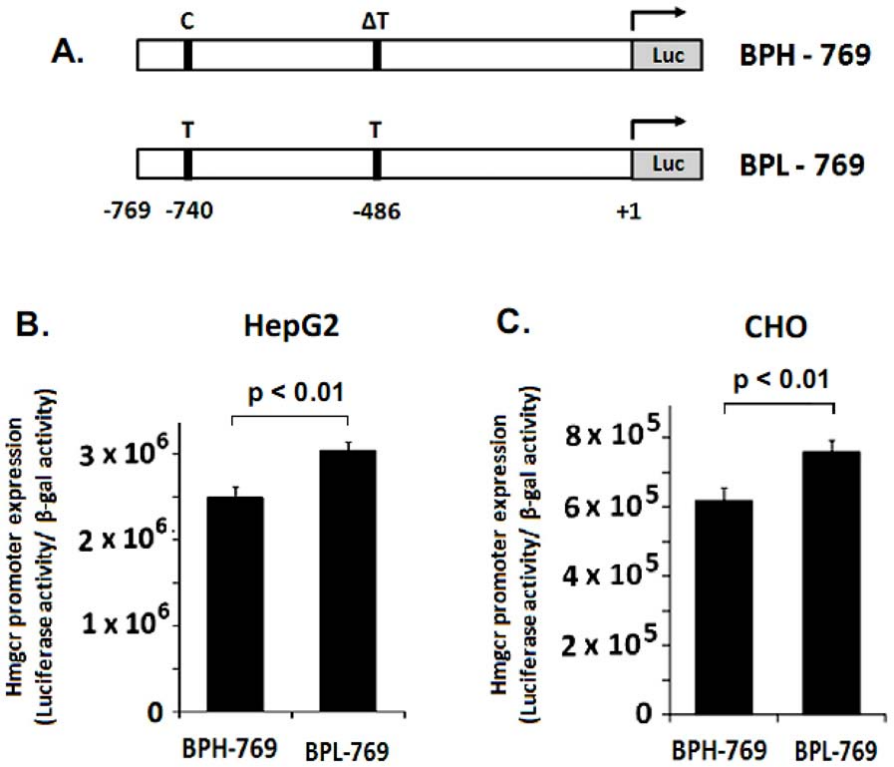
Role of the C-740T and ΔT-486T SNPs in the differential activity of *Hmgcr* promoter. The BPH-769 and BPL-769 promoter/firefly luciferase reporter constructs harboring C/T SNP at the −740 bp position and ΔT/T SNP at the −486 bp position (***panel A***) were generated as described in the Materials and Methods section. These constructs were transfected into HepG2 and CHO cells, along with co-transfected β-galactosidase expression plasmid. Cells were lysed 24–30 hrs after transfection and assayed for luciferase activity as well as β-galactosidase activity. Values shown in the bar graph are the Means ± S.E. of normalized luciferase activity (ratioed with respect to β-galactosidase activity) from at least three independent experiments. The BPL promoter displayed significantly higher activity than the BPH promoter in HepG2 (***panel B***) and CHO cells (***panel C***).

To assess the contribution of each *Hmgcr* promoter SNP towards the differential expression of transfected BPH and BPL promoter/reporter plasmids (Fig. 3), we undertook systematically progressive deletion of the promoter region. First, we generated the BPH-769 and BPL-769 constructs (Fig. 7A) wherein the C-874T SNP was excluded. Transfection of these constructs into HepG2 and CHO cells showed that the BPL promoter was ∼25–30% (p< 0.01) more active than the BPH promoter (Fig. 7B and 7C).

**Figure 8:**
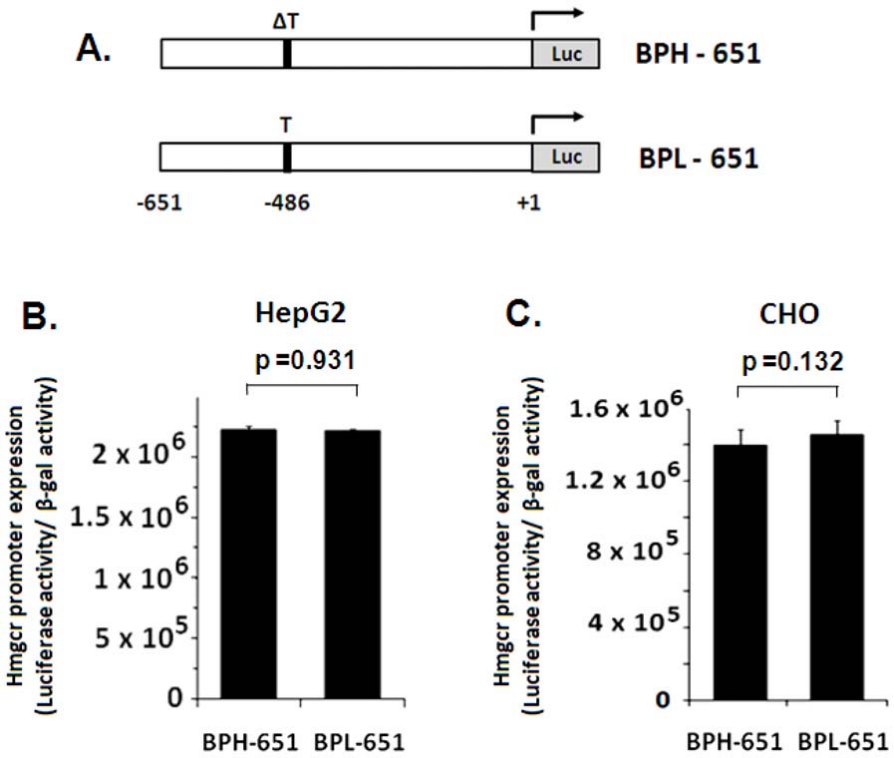
The ΔT-486T SNP in the mouse *Hmgcr* promoter is not functional. The BPH-651 and BPL-651 promoter/firefly luciferase reporter constructs harboring ΔT/T SNP at the -486 bp position (***panel A***) were generated as described in the Materials and Methods section. These constructs were transfected into HepG2 and CHO cells, along with co-transfected β-galactosidase expression plasmid. Cells were lysed 24–30 hrs after transfection and assayed for luciferase activity as well as β-galactosidase activity. Values shown in the bar graph are the Means ± S.E. of normalized luciferase activity (ratioed with respect to β-galactosidase activity) from at least three independent experiments. No difference in the activity between the BPH and BPL promoter was observed in HepG2 (***panel B***) and CHO cells (***panel C***).

Second, we generated the BPH-651 and BPL-651 constructs (Fig. 8A) wherein the C-874T and C-740T SNPs were excluded. Transfection of these constructs into HepG2 and CHO cells displayed no significant difference in *Hmgcr* promoter activity between BPH and BPL strains (Fig. 8B and 8C).

Taken together (Fig. 3, 7 and 8), while the −486 (T/ΔT) SNP did not influence promoter activity, the C-874T and C-740T SNPs were functional and responsible for the higher *Hmgcr* promoter activity in BPL than BPH. Consistently, the BPN-769 and BPH-769 constructs [that differed only at the −486 (T/ΔT) SNP position] did not display any difference in expression in HepG2 and CHO cells (data not shown). Likewise, the BPN-651 promoter-reporter construct also did not show any difference in luciferase activity as compared to BPL-651/BPH-651 construct in HepG2 and CHO cells (data not shown).

### The transcription factors c-Fos, n-Myc and Max modulate *Hmgcr* expression

**Figure 9:**
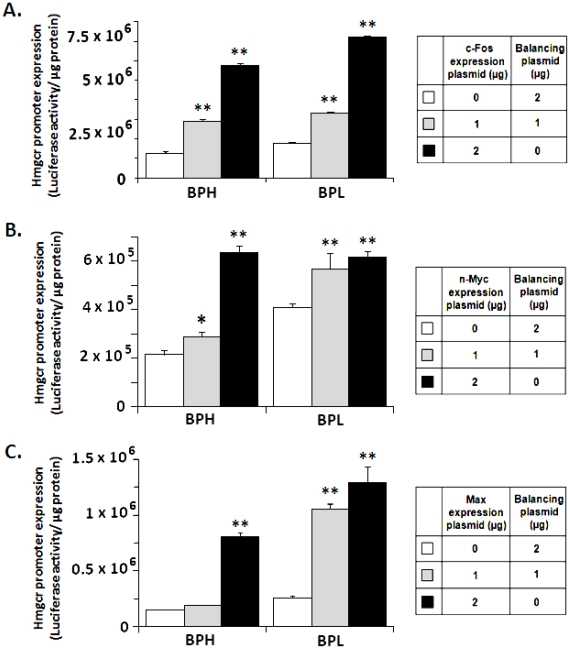
Activation of *Hmgcr* promoter activity by over-expression of c-Fos, n-Myc and Max. CHO cells were transiently transfected with increasing quantities (0–2.0 µg/well) of expression plasmids for the transcription factors c-Fos (***Panel A***), n-Myc (***Panel B***) or Max (***Panel C***) and 1.0 µg/well of BPL/BPH *Hmgcr* promoter (−961 to +43 bp)/firefly luciferase construct in 12-well cell culture plates. The total amount of plasmid DNA transfected to each well was made equal by using balancing amounts of the backbone plasmids (viz. pMiwSV plasmid in the cases of n-Myc/Max co-transfection and pSGI plasmid in the case of c-Fos co-transfection). Cells were lysed 24–30 hrs after transfection and assayed for luciferase activity. The results are expressed as ratios of firefly luciferase activity/µg protein and are the mean ± SE (n = 3–4 transfections for each construct). Although both BPL and BPH promoters displayed, *in general*, activation by each of these three transcription factors in a dose-dependent manner with respect to the control, the extents of activations were greater in case of BPL. (**) and (*) indicate p<0.01 and p<0.05 respectively with respect to the corresponding mock (i.e., without a co-transfected transcription factor). Co-transfection of Max expression plasmid resulted in more dramatic difference in promoter activities between BPL and BPH while the c-Fos expression plasmid showed the least difference in promoter activities, especially at the equimolar dose (1.0 µg/well transcription factor along with 1.0 µg/well promoter/reporter).

Since computational analysis of the promoter sequences around the C-874T and C-740T SNPs predicted better binding affinity of c-Fos, n-Myc and Max to the BPL promoter than the BPH promoter (Fig. 2), we tested the effect of co-transfection of expression plasmids of these transcription factors with BPL-/BPH- promoter-reporter constructs in CHO cells. The BPL- and BPH- promoters were differentially augmented by c-Fos/n-Myc/Max (Fig. 9). At the lower dose (1.0 µg/well) of the transcription factors, extents of activation of the BPL- promoter over BPH-promoter were ∼1.1-, ∼2.0- and ∼3.7–fold for c-Fos (Fig. 9A), n-Myc (Fig. 9B) and Max (Fig. 9C) respectively. At the higher dose (2.0 µg/well of c-Fos/n-Myc/Max), although no significant difference in stimulation between BPL- and BPH- promoter was observed in the case of n-Myc, the BPL-promoter activation was still significantly more than the BPH-promoter in case of c-Fos (∼1.4-fold, p<0.01; Fig. 9A) and Max (∼1.6-fold, p<0.01; Fig. 9C).

### Differential binding of nuclear proteins with BPL- and BPH- *Hmgcr* promoter domains

**Figure 10:**
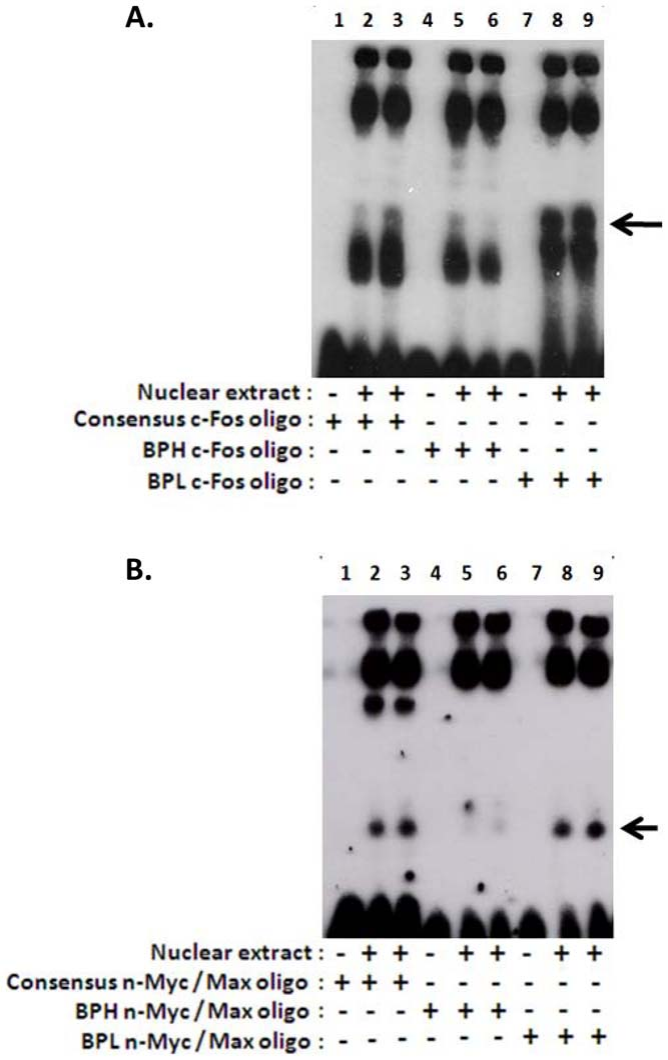
Electrophoretic mobility shift assays demonstrating complex formation between *Hmgcr* promoter domains and HepG2 nuclear proteins. ***Panel A:*** Oligos harboring the consensus c-Fos motif and the BPL-/BPH- *Hmgcr* promoter c-Fos motifs were biotinylated and incubated with HepG2 nuclear extracts as described in the Materials and Methods section. The BPL c-Fos oligo displayed dramatically enhanced formation a specific nuclear protein–oligo complex (lanes 8 and 9; indicated by a horizontal arrow) as compared to the BPH c-Fos oligo (lanes 5 and 6). As a negative control, no nuclear extract was added in some of the reactions (lane 1, consensus c-Fos oligo; lane 4, BPH c-Fos oligo; lane 7, BPL c-Fos oligo) to visualize the location of free/unbound probes on the gel. As a positive control, complexes formed by the consensus c-Fos oligo are shown in lanes 2 and 3. The results are representative of at least three separate experiments. ***Panel B:*** Oligos harboring the consensus n-Myc/Max motif and the BPL/BPH- *Hmgcr* promoter n-Myc/Max motifs were biotinylated and incubated with HepG2 nuclear extracts as described in the Materials and Methods section. The BPL n-Myc/Max oligo displayed formation of significantly higher amount of a specific nuclear protein–oligo complex (lanes 8 and 9; indicated by a horizontal arrow) as compared to the BPH n-Myc/Max oligo (lanes 5 and 6). As a negative control, no nuclear extract was added in some of the reactions (lane 1, consensus n-Myc/Max oligo; lane 4, BPH n-Myc/Max oligo; lane 7, BPL n-Myc/Max oligo) to visualize the location of free/unbound probes on the gel. As a positive control, complexes formed by the consensus n-Myc/Max oligo are shown in lanes 2 and 3. The results are representative of at least three separate experiments.

To test whether the BPL- and BPH- *Hmgcr* promoter domains harboring putative binding sites for c-Fos and n-Myc/Max interact differently with HepG2 nuclear proteins, we carried out EMSA experiments. The BPL c-Fos oligo (that contained a better binding site for c-Fos) showed altered complex formation pattern as compared to the BPH c-Fos oligo (Fig. 10A, lanes 8 and 9 *vs.* lanes 5 and 6). Likewise, the BPL n-Myc/Max oligo (containing a better binding site for n-Myc/Max) yielded higher amount of a specific nuclear protein-oligo complex than the BPH n-Myc/Max oligo (Fig. 10B, lanes 8 and 9 *vs.* lanes 5 and 6). As a positive control, we tested complex formation using consensus c-Fos oligo (Fig. 10A, lanes 2 and 3) and consensus n-Myc/Max oligo (Fig. 10B, lanes 2 and 3). As negative controls, when no nuclear extract was added to binding reactions, only labeled oligo bands were observed (Fig. 10A and 10B: lanes 1, 4 and 7).

## Discussion

### Mouse *Hmgcr* genetic polymorphisms

**Figure 11:**
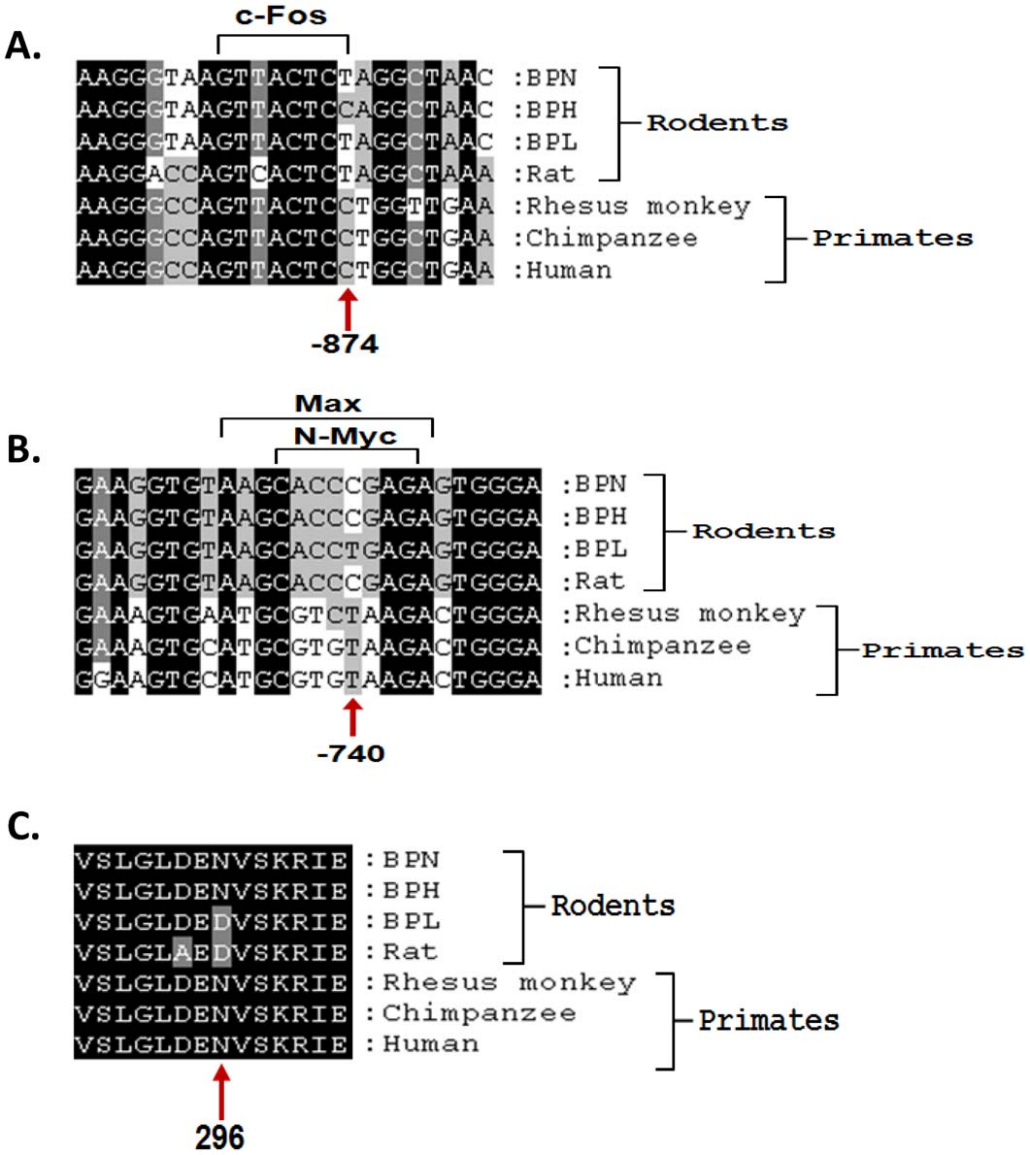
Conservation of Hmgcr sequences among mammalian species. Alignment of orthologous sequences around the promoter polymorphisms C-874T (***panel A***), C-740T (***panel B***) and the amino acid variant N296D (***panel C***) in mouse *Hmgcr* was carried out using Clustal W. The promoter as well as protein sequences were found to be highly conserved among the mammals. The binding motifs for the transcription factors c-Fos (***panel A***) and n-Myc/Max (***panel B***) are shown within brackets. The BPH, BPL and BPN sequences were determined in this study (as described in the Materials and Methods section) while the other sequences were obtained from UCSC/NCBI public databases; the accession numbers for the promoter sequences are: human, NM_000859.2; chimpanzee, XM_001148324.1; rhesus monkey, XM_001104607.2; rat, NM_013134.2, and the accession numbers for the amino acids sequences are: human, NP_000850.1; chimpanzee, XP_001148324.1; rhesus monkey, XP_001104607.1; rat, NP_037266.2). The positions of the nucleotide/amino acid variations are indicated by upward arrows.

Several SNPs were discovered in the mouse *Hmgcr* gene (Table 2). Two (C-874T and C-740T) of the 3 promoter SNPs that distinguished BPL from BPH were transcriptionally active. Alignment of the orthologous mammalian sequences around these two SNP positions revealed that these promoter regions were highly conserved (Fig. 11). The C allele at −874 bp (present in BPH) occurred in primates while the −874T allele was found in rodents (Fig. 11A). On the other hand, the C allele at −740 bp (present in BPH) occurred in rodents while the corresponding T allele was found in primates (Fig. 11B).

Seven of the 8 exonic SNPs altered amino acid residues while one was synonymous. Notably, two non-synonymous variations (Aspartate296Asparagine in the trans-membrane region and Glutamic acid455Lysine in the catalytic domain of the enzyme) distinguished BPL from BPH. Interestingly, alignment of the orthologous sequences around the non-synonymous SNPs revealed that while the *Hmgcr* coding sequence was extremely conserved among mammals (data not shown) amino acid variations discovered in this study were unique to these strains excepting the BPL-Aspartate296, which also occurred in rat (Fig. 11C). However, the functional implications of these polymorphisms are not yet known.

Besides the promoter and 20 exons, we also sequenced the exon-intron borders of the *Hmgcr* gene to probe for any alternative splicing among these strains because a number of studies reported variations in *HMGCR* exon/intron splicing in humans and CHO cells [34]–[39]. In humans, *HMGCR* alternative splicing has been associated with inter-individual variation in plasma low-density lipoprotein cholesterol response to statin treatment [34], [35]. However, we did not find any interstrain variation in the consensus splice donor (GU) or acceptor (AG) dinucleotides in the *Hmgcr* introns.

### Molecular basis of altered *Hmgcr* expression

Expression of promoter-reporter constructs in cultured cells showed that the C-874T and C-740T SNPs were functional. The −740T allele alone contributed to ∼25–30% higher promoter activity than the −740C allele (Fig. 7 and 8); the −740T and −874T alleles together resulted in ∼150–180% higher expression of the BPL promoter than the BPH promoter (that contained the −740C and −874C alleles) (Fig. 3). Consistently, the BPN promoter (that harboured the −740C and −874T alleles) displayed intermediate activity (∼125–135% as compared to the BPH promoter; Fig. 3).

How might the C-874T and C-740T SNPs alter the *Hmgcr* promoter activity? Computational analysis (by ConSite) revealed that these T alleles offered better binding sites for c-Fos, n-Myc and Max as compared to the C alleles (Fig. 2). Consistent with these computational predictions, over-expression of c-Fos, n-Myc and Max in CHO cells augmented the BPL-*Hmgcr* promoter activity to a greater extent than the BPH-*Hmgcr* promoter (Fig. 9). Interestingly, the extent of alteration of the promoter expression by c-Fos/n-Myc/Max paralleled with the differences in ConSite score (an index of binding affinity of a transcription factor protein with a DNA motif) between the motifs for BPL and BPH. For example, among these three proteins, the difference in ConSite score was the highest between BPL-Max and BPH-Max motifs (7.054–2.925≈4 units) and the least between BPL-c-Fos and BPH-c-Fos motifs (3.933–2.994≈1 unit); Max co-expression resulted in more dramatic differences between BPH and BPL promoter activity while the c-Fos co-expression showed the least difference in promoter activities, especially at the lower doses of the transcription factor plasmid (Fig. 9). In addition, EMSA experiments also showed enhanced complex formation of HepG2 nuclear proteins with the BPL c-Fos/n-Myc/Max oligos than the BPH c-Fos/n-Myc/Max oligos (Fig. 10) confirming roles of the promoter variants in the differential *Hmgcr* gene expression.

Of note, c-Fos, a basic leucine zipper (bZIP) protein and a major component of the activator protein-1 transcription factor complex, has been implicated as a regulator of cell proliferation, differentiation and transformation [40]–[42]. A recent study reported recruitment of c-Fos to the *Hmgcr* promoter for transcriptional regulation under acute kidney injury in mice [43]. The transcription factor n-Myc is a basic helix-loop-helix leucine zipper (bHLH-ZIP) protein that heterodimerizes with the transcription factor Max and play important roles in neuronal differentiation and cell proliferation [44]–[48]. Although n-Myc and Max have been reported to regulate transcription of many genes [49]–[53], this study, for the first time, provides evidence for regulation of the *Hmgcr* gene by these transcription factors.

### Differential regulation of *Hmgcr* promoters by nicotine and cholesterols

Nicotine caused dose-dependent activation of BPL and BPH promoters in both HepG2 and CHO cells; the effect was more prominent in the case of BPL (Fig. 5). Are there functional nicotinic acetylcholine receptors (nAChRs) in these cell types to elicit nicotine-induced *Hmgcr* promoter stimulation? In isolated rat hepatocytes, nicotine increased intracellular calcium concentration and this phenomenon was blocked by *d*-tubocurarine, a nAChR antagonist [54]. A recent study also detected the presence of alpha-7 nAChRs in mouse liver by utilizing two carbon-11-labeled alpha-7 nAChR agonists [55]. On the other hand, whereas the presence of α7, α4 or β2 subunits of nAChR in CHO cells could not be detected [56], the existence of an intraovarian, non-neuronal cholinergic system in human and rat has been reported [57], [58]. In view of these reports, we speculate that the nicotinic stimulation of *Hmgcr* promoter in HepG2 and CHO may be mediated by some nAchR subtype(s). Further studies are required to establish the mechanism of nicotinic signal transduction in these cells.

The differential nicotinic activation of *Hmgcr* promoters may be mediated by altered interaction of the c-Fos/n-Myc motifs *in cis* with c-Fos and n-Myc *in trans* since these BPH- and BPL- promoter motifs have different binding affinities with these nuclear proteins (Fig. 2 and 10). Notably, several studies have shown that nicotine regulates expression of c-Fos and Myc in different cell types [59]–[62]. The different extent of nicotinic stimulation of the *Hmgcr* promoters may also be contributed by the transcription factor cyclic AMP response element binding protein (CREB) because nicotine is known to activate the phosphorylation of CREB, which induces the expression its early target c-Fos [63], [64]. Thus, our initial findings indicate hitherto un-described involvement of several transcription factors in nicotine-evoked activation of *Hmgcr* transcription and hence their possible roles in nicotinic modulation of cholesterol biosynthesis. However, further studies are required to confirm the contribution of these transcription factors in the up-regulation of *Hmgcr* expression by nicotine.

Similar to nicotine, the BPL- and BPH- *Hmgcr* promoters responded differentially to cholesterols, albeit the effect was down-regulation of gene expression; BPL-promoter showed more pronounced repression of the luciferase activity than the BPH-promoter (Fig. 6). What might be the molecular mechanism for this differential response by these promoters? Although the identities of the transcription factors involved in the sterol regulation of *Hmgcr* promoter still remain incompletely understood, previous studies demonstrated important roles for the sterol regulatory element (SRE-1) SRE binding proteins (SREBPs), CCAAT-binding factor/nuclear factor-Y (CBF/NF-Y) and CREB [65]–[68]. Since the BPL- and BPH- *Hmgcr* did not differ at the SRE-1 or CBF/NF-Y or CRE motifs and the differential expression under the basal conditions was mediated by c-Fos/n-Myc/Max (Fig. 2, 9, 10), the greater sterol-repression (i.e. negative feedback regulation) of the BPL- promoter may be modulated by interaction of SREBP/CBF/NF-Y/CREB with c-Fos/n-Myc/Max. Of note, 25-hydroxycholesterol has been reported to cause calcium-dependent activation of c-Fos via the ERK1/2 signaling pathway in monocytic THP-1 cells [69]. Further studies are required to unravel the possible regulatory role of c-Fos/n-Myc/Max in repression of *Hmgcr* expression by cholesterols.

### *Hmgcr* endogenous gene expression studies: possible mechanisms for differential transcript abundance

Tissue distribution pattern of the *Hmgcr* gene showed a high level of expression in the liver [70]. Consistently, the Hmgcr protein level was reported to be 4- to 6 -fold higher in the liver than most peripheral tissues [27]. In this study, we measured the abundance of *Hmgcr* transcripts in BPH and BPL liver tissues and detected as much as ∼3.4-fold higher expression in BPL (Fig. 4). Intriguingly, such a large magnitude *Hmgcr* over-expression in BPL liver cannot be accounted for by functional promoter variants alone because the transfected BPL promoter showed only up to ∼1.8-fold higher activity than the BPH promoter in HepG2 cells (Fig. 3). Other genetic and/epigenetic factors may, therefore, also contribute to the difference in the endogenous *Hmgcr* expression in these mouse models. However, the higher *Hmgcr* expression in BPL is consistent with the elevated hepatic cholesterols (∼1.4-fold, p = 0.025) and plasma cholesterols (∼1.3-fold, p<0.01) in these mice as compared to BPH mice (Mouse Phenome Database, Jackson Laboratory; <www.jax.org/phenome>).

Of note, in an earlier genome-wide transcriptome profiling study in adrenal glands, BPH mice displayed ∼3-fold higher *Hmgcr* expression than BPL mice [12]. What might be the mechanism of such directionally opposite differential *Hmgcr* expression between liver and adrenal glands in these mice? Given that in both these studies, BPL and BPH mice of similar age (5-7 weeks old) and same sex (male) were used, the altered *Hmgcr* expression might be caused/contributed by other factors. For example, *Hmgcr* expression in these strains might occur in a tissue-specific manner, perhaps mediated by specific transcription factors in liver versus adrenals tissues, as reported in the cases of human and rat orthologues of *Hmgcr* as well as other genes [19], [71]–[76]. Further, the concentration of cholesterol in the plasma reflects the net contribution of cholesterol synthesis, secretion, and absorption from various tissues, including the liver and adrenal glands [77]. Although extrahepatic tissues also have local cholesterol biosynthesis systems [78]–[80], the cholesterol needs of several tissues are mainly met by receptor-mediated uptake of low-density lipoprotein from the circulation [32]. Notably, in female rats, the highest rate of uptake of sterols was found in adrenal glands where only 4% of the tissue sterol content came from local synthesis [81]. In BPH mice, the supply of low-density lipoprotein cholesterol to the extrahepatic tissues including adrenals might be insufficient due to lower circulating cholesterol in this strain. Therefore, the upregulation of *Hmgcr* expression in BPH adrenals [12] might take place to increase *de novo* cholesterol synthesis in order to meet the local sterol-demand.

Thus, the BPH mouse appears as a unique model where the dyslipidemia and hypertension are not inter-connected. Given that these genetically hypertensive mice were derived solely based on high BP phenotype [14], this phenomenon might be caused/contributed by segregation of genes. However, such dissociation between severe hypertension and enhanced lipid synthesis in this model may yield important insights into factors that govern the coupling of these traits in humans. Interestingly, this is reminiscent of the recent findings on chromogranin A gene knockout mice, which displayed severe hypertension but unaltered plasma cholesterol level as compared to wild type mice [82].

### Concluding remarks

The present study unfolded the quantitative impact of two *Hmgcr* promoter SNPs on gene expression in three mouse models of human essential hypertension. The functional implication of these SNPs for hypertension remains to be elucidated. Further investigations are also required to unravel the qualitative impact of non-synonymous SNPs (especially those in the catalytic region). Nonetheless, this is the first report on identification and molecular characterization of functional polymorphisms at the *Hmgcr* locus in genetically hypertensive versus genetically hypotensive mice. Future studies may establish additional genetic and molecular links of the *Hmgcr* gene to hypertension and associated cardiovascular disease states.

## Supporting Information

Text S1 **Supplementary methods.**
(DOC)

Table S1 **Basal expression of** ***Hmgcr*** **promoter-luciferase reporter plasmids in cultured cells.**
(DOC)
