# Supplementary material for: Functional Promoter Polymorphisms Govern Differential Expression of HMG-CoA Reductase Gene in Mouse Models of Essential Hypertension
Source: PLoS One. 2011 Jan 31;6(1):e16661. doi: 10.1371/journal.pone.0016661 (PMC3031630; doi:10.1371/journal.pone.0016661)
Supplement: Text S1 — Supplementary methods. (DOC) [file pone.0016661.s001.doc]

**Supporting Information S1.**

**SUPPLEMENTARY METHODS.**

**Real-time PCR for quantification of endogenous *Hmgcr* expression.**

About 5 μg of each RNA sample was subjected to total cDNA synthesis by using ProtoScript Moloney Murine Leukemia Virus [M-MuLV] Taq RT-PCR kit (New England Biolabs, USA). Twenty microliter of the cDNA reaction was diluted to 50 µl in nuclease-free water and stored at -20 ^0^C until use. Absence of genomic DNA contamination in the cDNA preparation was confirmed by a test PCR using *Hmgcr* PCR primers from the coding exons 9 and 11: (upstream primer, [+11481bp] 5’-TTGGTCCTTGTTCACGCTCATAG-3’ [+11503 bp] in exon 9 and downstream primer, [+12138 bp] 5’-CTCTGCTTGTAGTCTCTGCTTCCAC-3’ [+12114 bp] in exon 11; the numbers are with respect to the transcription initiation site as +1). The primer pair was designed in such a way that pure cDNA would result in a 447 bp product, whereas genomic DNA contamination, if any, in the cDNA preparation would amplify a higher molecular size (viz. 657 bp) product containing an additional 210 bp segment (comprising of 98 bp intron 9 and 112 bp intron10 regions). As a negative control, no PCR product was obtained when cDNA was not first prepared from the mRNA by reverse transcriptase treatment indicating absence of any contaminating genomic DNA with the mRNA preparations. As a second negative control, no PCR product was obtained when water was added instead of cDNA preparations into the reaction mixture. On the other hand, genomic DNA of BPL and BPH mice yielded ~650 bp bands.

Real-time PCR was carried out using 0.5 μl of cDNA preparation per 10 μl reaction and DyNAmo^TM^ HS SYBR® Green qPCR Kit (Finnzymes, USA) in an Eppendorf Mastercycler® ep realplex Thermal Cycler. Specific primers to amplify a 118 bp region of the mouse *Hmgcr* exon 10 were used. For normalization of the endogenous *Hmgcr* gene expression, a 124 bp GAPDH and a 151-bp amplicon of 18S rRNA (as a house-keeping controls) were amplified under identical conditions. The PCR program used for the real-time reaction was: 95^0^C for 2 min; 95^0^C for 15 sec, 68^0^C for 20 sec for 40 cycles.

**Reporter assays.**

Cells were lysed 24-30 hours after transfection with 300 µl/well of lysis buffer [0.1 M phosphate buffer (K_2_HPO_4_+ KH_2_PO_4_) pH 7.8, 1 mM DTT and 0.1% Triton-X 100] and the ﬁreﬂy luciferase and β-galactosidase activities in the cell lysates were measured. For luciferase assay, luminescence in 80 µl of transfected cell lysates were determined as relative light unit (RLU) using the Sirius FB12 luminometer (Berthold, Germany) after addition of 100 µl of assay buffer [100 mM Tris-acetate pH 7.8, 10 mM Mg-acetate, 1 mM EDTA pH 8.0, 3 mM ATP (Hi-Media, India) and 100 µM beetle luciferin K^+^-salt (Promega, USA)]. The beta-gal assay was carried out using 150 µl of heat-inactivated cell lysate that was mixed with 150 µl of 2x assay buffer [200 mM phosphate buffer pH 7.3, 2 mM MgCl_2_, 100 mM β-mercaptoetanol, 1.33 mg/ml of ortho-nitrophenyl β-D-galactopyranoside (Sigma-Aldrich, USA)]. The tubes were incubated at 37 ^0^C for 30 min or until the color of reaction mixture changed to faint yellow. Reactions were stopped by adding 500 µl of 1M sodium carbonate and absorbances were measured at 420 nm. The results were expressed as the ratios of ﬁreﬂy luciferase/β-galactosidase activity.

**Preparation of nuclear extracts.**

Nuclear protein extracts from HepG2 and CHO cells were prepared using ProteoJET cytoplasmic and nuclear protein extraction kit (Fermentas Life Sciences, USA). In brief, ~ 5 x 10^6^ cells were harvested in 1x phosphate-buffered saline and pelleted at 250 x g for 5 min at 4^0^C. The cells were lysed with 200 µl of chilled lysis buffer [supplemented with 1 mM DTT and protease inhibitors (protease inhibitor cocktail, Sigma-Aldrich, USA)]. After a 10 min incubation on ice, the nuclei were separated by centrifugation at 500 x g for 7 min at 4^0^C and washed twice with 500 µl of nuclei wash buffer. The nuclei were suspended in 150 µl of nuclei storage buffer (supplemented with 20 mM DTT and protease inhibitor cocktail) and lysed with 15 µl of nuclei lysis buffer with shaking for 15 min at 4 ^0^C. The nuclear lysates were cleared by centrifugation at 20000 x g for 5 min at 4 ^0^C and stored in aliquots at -80^0^C until use. The total protein amounts in these extracts were determined using Bradford’s assay reagents (Sigma-Aldrich, USA).

**Labeling of the EMSA primers.**

Single stranded oligomers were biotinylated using Biotin 3’ End Labeling kit (Pierce, USA) as per manufacturer’s protocol. In brief, 3’ ends of single stranded DNA oligomers (at 5 µM concentration) were labeled (by incubation at 37^0^C for 30 min) with biotin-11 dUTP (500 nM) using 10 units of TdT (Terminal deoxynucleotidyl transferase). The labeling reactions were terminated with 10 µM of EDTA. The oligos were purified by extraction with equal volumes of chloroform: Iso-amyl alcohol mixture (24:1). Equal amount of each oligomer and its complementary strands were annealed to a final oligomer concentration of 50 nM. Efficiency of the biotin labeling of the primers was tested by dot blot of labeled oligomers and densitometric analysis.
