# Supplementary material for: Functional Promoter Polymorphisms Govern Differential Expression of HMG-CoA Reductase Gene in Mouse Models of Essential Hypertension
Source: PLoS One. 2011 Jan 31;6(1):e16661. doi: 10.1371/journal.pone.0016661 (PMC3031630; doi:10.1371/journal.pone.0016661)
Supplement: Table S1 — Basal expression of Hmgcr promoter-luciferase reporter plasmids in cultured cells. (DOC) [file pone.0016661.s002.doc]

**Table S1.** Basal expression of *Hmgcr* promoter-luciferase reporter plasmids in cultured cells.*

| Promoter  Cell line | BPH promoter activity  (luciferase/beta-gal) | BPL promoter activity  (luciferase/beta-gal) | BPN promoter activity  (luciferase/beta-gal) |
| --- | --- | --- | --- |
| **HepG2** | 3319563±481424 | 5787873±23398 | 4263007±30337 |
| **CHO** | 1790501±222497 | 2750723±27199 | 2411318± 81750 |
| **HEK-293** | 8997634±529427 | 13002644±542650 | 19881396±445681 |
| **N2A** | 1393887±135637 | 2637241±89918 | 1958527±198300 |

*Promoter reporter constructs harboring ~ 1 kb *Hmgcr* promoter region of BPH/ BPL/BPN mouse were transfected to the cell lines, along with the co-transfected control plasmid pCMV-βGal. The cells were assayed for luciferase and β-galactosidase activities 24-30 hrs after transfection. Values shown are the Means ± S.E. of normalized (ratioed) luciferase activity to β-galactosidase activity from at least three independent experiments. The transfected promoters displayed signiﬁcantly different activities among three strains in HepG2 (p < 0.01), CHO (p < 0.01), HEK-293 (p < 0.0001) and N2A (p=0.0031) cells as determined by one-way ANOVA with Tukey-Kramer multiple comparisons post-test. In general, the transfected BPL promoter was more active than the BPH promoter while the BPN promoter displayed intermediate expression.
